# Differential effects of cytokines and corticosteroids on Toll-like receptor 2 expression and activity in human airway epithelia

**DOI:** 10.1186/1465-9921-10-96

**Published:** 2009-10-16

**Authors:** Audra A Winder, Christine Wohlford-Lenane, Todd E Scheetz, Brie N Nardy, Lori J Manzel, Dwight C Look, Paul B McCray

**Affiliations:** 1 Department of Pediatrics, Carver College of Medicine, University of Iowa, Iowa City, IA USA; 2 Department of Opthalmology, Carver College of Medicine, University of Iowa, Iowa City, IA USA; 3 Department of Internal Medicine, Carver College of Medicine, University of Iowa, Iowa City, IA USA; 4 Department of Microbiology, Carver College of Medicine, University of Iowa, Iowa City, IA USA; 5 College of Engineering, University of Iowa, Iowa City, IA USA

## Abstract

**Background:**

The recognition of microbial molecular patterns via Toll-like receptors (TLRs) is critical for mucosal defenses.

**Methods:**

Using well-differentiated primary cultures of human airway epithelia, we investigated the effects of exposure of the cells to cytokines (TNF-α and IFN-γ) and dexamethasone (dex) on responsiveness to the TLR2/TLR1 ligand Pam3CSK4. Production of IL-8, CCL20, and airway surface liquid antimicrobial activity were used as endpoints.

**Results:**

Microarray expression profiling in human airway epithelia revealed that first response cytokines markedly induced TLR2 expression. Real-time PCR confirmed that cytokines (TNF-α and IFN-γ), dexamethasone (dex), or cytokines + dex increased TLR2 mRNA abundance. A synergistic increase was seen with cytokines + dex. To assess TLR2 function, epithelia pre-treated with cytokines ± dex were exposed to the TLR2/TLR1 ligand Pam3CSK4 for 24 hours. While cells pre-treated with cytokines alone exhibited significantly enhanced IL-8 and CCL20 secretion following Pam3CSK4, mean IL-8 and CCL20 release decreased in Pam3CSK4 stimulated cells following cytokines + dex pre-treatment. This marked increase in inflammatory gene expression seen after treatment with cytokines followed by the TLR2 ligand did not correlate well with NF-κB, Stat1, or p38 MAP kinase pathway activation. Cytokines also enhanced TLR2 agonist-induced beta-defensin 2 mRNA expression and increased the antimicrobial activity of airway surface liquid. Dex blocked these effects.

**Conclusion:**

While dex treatment enhanced TLR2 expression, co-administration of dex with cytokines inhibited airway epithelial cell responsiveness to TLR2/TLR1 ligand over cytokines alone. Enhanced functional TLR2 expression following exposure to TNF-α and IFN-γ may serve as a dynamic means to amplify epithelial innate immune responses during infectious or inflammatory pulmonary diseases.

## Background

The airway epithelium plays an important role in orchestrating pulmonary innate and adaptive immune responses. This mucosal surface is a site of first contact with the environment and has evolved many mechanisms to recognize and respond to inhaled or aspirated microorganisms. Epithelial responses to microbes are initiated via pattern recognition receptors including the Toll-like receptors (TLRs) [1]. TLRs are a family of 10 receptors in humans that recognize a variety of microbial molecular patterns and regulate immune responses. Airway epithelial cell responses to a number of TLRs, including TLR2 [2-4], TLR3 [5], TLR4 [6], TLR5 [7,8], and TLRs 6 through 9 [9,10] have been investigated in human cells and animal models. TLR activation initiates signaling that culminates in a number of host defense responses including the secretion of antimicrobial peptides, cytokines, and chemokines by epithelial cells [11,12].

TLR expression is regulated in a cell and tissue specific manner [11,13-15]. Experimental evidence in humans [16] and animal models [17-20] indicates that the expression and function of several TLRs is developmentally regulated. Here we focus on TLR2 expression and function in well-differentiated human respiratory epithelia. TLR2 forms heterodimers with either TLR6 or TLR1; these heterodimers recognize diacyl and triacyl lipopeptides, respectively [21,22]. TLR2 signaling occurs via a MyD88-dependent pathway leading to the nuclear translocation of NF-κB [14], and induction of various inflammatory cytokines, including IL-8, and antimicrobial peptides, such as human beta-defensin 2 (HBD-2) [4,23].

We hypothesized that first response cytokines would influence both the array of functional TLRs and their responses to stimuli. Further, dexamethasone, when co-administered with pro-inflammatory cytokines, was reported to synergistically enhance TLR2 expression in the human alveolar and bronchial epithelial cell lines A549 [24] and BEAS-2b [3]. We therefore investigated the effects of both cytokine and glucocorticoid exposure on TLR2 expression in primary cultures of human airway epithelia.

## Methods

### Culture of human airway epithelia

Primary cultures of human airway epithelia were grown at an air-liquid interface as described previously [25]. Human donor lungs were obtained from individuals without primary pulmonary diseases whose lungs were determined to be unsuitable for organ transplantation. The use of human tissues was approved by the University of Iowa Institutional Review Board. Well-differentiated (> 2 weeks in culture) tracheal and bronchial epithelia were used in all studies. Epithelia were maintained in DMEM/F-12 with 1% penicillin-streptomycin, 50 μg/ml gentamicin, and 2% Ultroser G. In a microarray experiment (described below), epithelia were incubated for 24 hours in 2% Ultroser G media containing 100 ng/ml each of recombinant interleukin-1-beta (IL-1β; Sigma, St. Louis, MO), tumor necrosis factor-alpha (TNF-α; Sigma), and interferon-gamma (IFN-γ; Sigma). For all other experiments, epithelia were placed in serum free DMEM/F-12 for 48 hours, then incubated overnight (18 hr) in media containing cytokines (100 ng/ml each of TNF-α and IFN-γ), 1 μM water-soluble dexamethasone (D2915; Sigma), cytokines plus dexamethasone, or control serum-free media (100 μl volume applied apically, and 500 μl basolaterally). Cell viability was similar under all experimental conditions.

In TLR2 receptor agonist experiments, epithelia were rinsed with media, then incubated for an additional 24 hours in media containing 25 μg/ml Pam3CSK4 (tlrl-pms; InvivoGen, San Diego, CA), a synthetic bacterial lipoprotein TLR2/TLR1 ligand, or control serum-free media. Where specified, media containing cytokines ± dexamethasone or Pam3CSK4 were applied to only the apical or basolateral surface, with control serum-free media applied contralaterally. Prior to assays of airway surface liquid antimicrobial activity (see Antimicrobial Assays, below), epithelia were incubated for 5 days in antibiotic-free media and washed daily with antibiotic-free media to remove residual antibiotics.

### RNA isolation and quantitative reverse transcription-PCR (RT-PCR)

RNA was extracted using an RNeasy Mini Kit (Qiagen Inc., Valencia, CA) according to manufacturer's protocol. For each sample, 1 μg of total RNA was used as a template for first-strand cDNA synthesis. Quantitative PCR (ABI 7900) was used to amplify the TLR or HBD-2 PCR products along with GAPDH transcripts in a single reaction. Forward and reverse primers and TaqMan probes were designed using Primer Express software (P-E Applied Biosystems, Foster City, CA). Primers and probes were: TLR2 forward, 5'-GGCCAGCAAATTACCTGTGTG-3'; TLR2 reverse, 5'-AGGCGGACATCCTGAACCT-3'; TLR2 probe, 5'-TCCATCCCATGTGCGTGGCC-3'; TLR1 forward, 5'-CAGTGTCTGGTACACGCATGGT-3'; TLR1 reverse, 5'-TTTCAAAAACCGTGTCTGTTAAGAGA-3'; TLR1 probe, 5'-TGCCCATCCAAAATTAGCCCGTTC-3'; TLR6 forward, 5'-GAAGAAGAACAACCCTTTAGGATAGC-3'; TLR6 reverse, 5'-AGGCAAACAAAATGGAAGCTT-3'; TLR6 probe, 5'-TGCAACATCATGACCAAAGACAAA-3'; HBD-2 forward, 5'-CCTGTTACCTGCCTTAAGAGTGGA-3'; HBD-2 reverse, 5'-ACCACAGGTGCCAATTTGTTTA-3'; HBD-2 probe: 5'-CCATATGTCATCCAGTCTTTTGCC-3'. The TLR and HBD-2 probes were labeled with the fluorophore FAM, and the GAPDH probe with the fluorophore JOE. *C*_*T *_for the TLR or HBD-2 PCR product was normalized against the *C*_*T *_for GAPDH.

### Microarray hybridization

Five micrograms of total RNA was processed using the Affymetrix GeneChip one-cycle target labeling kit (Affymetrix, Inc., Santa Clara, CA) following the manufacturer's protocols. The resultant biotinylated cRNA was hybridized to a custom GeneChip Human Airway Array (HsAirway, Affymetrix, Inc.). Tracheal and bronchial epithelial cells from seven donors were used in this study. The custom Affymetrix array (HsAirway) was comprised of approximately 23,000 probe sets derived from sequencing of cDNA libraries prepared from human lung, primary airway epithelial cells, and human alveolar macrophages [26]. The arrays were washed, stained, and scanned using the Affymetrix Model 450 Fluidics Station and Affymetrix Model 3000 scanner. Each sample and hybridization underwent quality control evaluation, including cRNA amplification of more than 4-fold, percentage of probe sets reliably detecting between 40 and 60 percent present call, and 3'-5' ratio of GAPDH gene less than 3.

The hybridizations were normalized using the RMA (robust multi-chip averaging) [27] method from Bioconductor [28] to obtain summary expression values for each probe set. Gene expression levels were analyzed on a logarithmic scale. Differentially expressed genes were identified using a 1-factor ANOVA test. Heat-map visualizations were generated with GenePattern [29]. Global scaling of the expression levels was used to allow experiment-wide comparison of gene expression.

### Protein quantification by ELISA

IL-8 and CCL20 protein abundance in the basolateral media was measured by ELISA (Duoset DY208 (IL-8) and DY360 (CCL20); R&D Systems, Minneapolis, MN).

### Antimicrobial assays

Apical washings from epithelia were obtained after a 24-hour incubation with the TLR2 agonist Pam3CSK4 or control serum-free, antibiotic-free media. Airway surface liquid (ASL) was obtained by adding 100 μl of sterile 1× PBS to the apical surface and immediately pipetting off the fluid. We used a modified radial diffusion assay to quantify ASL antimicrobial activity as described previously [30]. Briefly, 4 × 10^6 ^bacteria in mid-log phase were suspended in an underlay gel. 2.5 mm diameter wells were punched into the gel and filled with 0.04-5 μl of ASL, with 0.02% acetic acid with 0.1% human serum albumin (Sigma) to equal 5 μl, or control antibiotic (gentamicin, 0.4-50 μg/ml). The plates were then incubated for 3 hours at 37°C. Nutrient rich gels were then overlaid, and the plates incubated at 37°C overnight. Zones of clearance were manually measured and plotted on a semi-log graph where the X-intercept represents the minimal inhibitory concentration (MIC) [31]. Test organisms included *Escherichia coli *DH5α, *Pseudomonas aeruginosa *PA01, and a clinical strain of *Listeria monocytogenes*.

### Immunoblot Analysis

Whole cell protein extract preparation and immunoblot analysis were performed as described previously [32,33]. Primary antibodies used to detect specific cellular and nuclear proteins were: mouse IgG1 mAb clone L35A5 against human IκBα, rabbit polyclonal IgG 9171 against human Stat1 phosphorylated on tyrosine-701, rabbit polyclonal IgG 9172 against human total Stat1, rabbit IgG mAb clone 3D7 against human p38 MAP kinase phosphorylated on threonine-180 and tyrosine-182, rabbit IgG mAb clone 7D6 against human total p38 MAP kinase from Cell Signaling Technology (Beverly, MA); mouse IgG2α mAb clone AC-74 against human β-actin from Sigma-Aldrich (St. Louis, MO); rabbit polyclonal antiserum against human heat shock protein (HSP)-90 from Assay Designs (Ann Arbor, MI). Primary antibody binding was detected using goat antirabbit or antimouse IgG conjugated to horseradish peroxidase (Santa Cruz Biotechnology, Santa Cruz, CA or Cell Signaling Technologies) and an enhanced chemiluminescence detection system (Amersham Biosciences, Uppsala, Sweden). Reprobing of membranes was done after washing twice in Restore™ buffer (Pierce, Rockford, IL) for 15 min at 37°C. In some experiments, radiographic film images were analyzed using ImageJ software [34]. To generate an integrated density level, band area was multiplied by the band mean gray value, and the integrated density for IκBα or phosphorylated p38 was divided by the corresponding β-actin or HSP-90 level creating a ratio for each sample.

### NF-κB Activation Assay

NF-κB-dependent gene activation was determined using a recombinant adenoviral vector that expresses a luciferase gene driven by four tandem NF-kB enhancer sequences as described previously [32,35]. *Photinus pyralis *luciferase activity was determined using a commercial luciferase reporter assay kit (Promega) and a Lumat LB 9501 luminometer (Berthold, Bad Wildbad, Germany).

### Statistical Analysis

Assessment of statistical significance for quantitive PCR and ELISA data was performed using one-tailed Student's t tests with Microsoft Excel. P values < 0.05 were considered significant. Luciferase assays and densitometry analysis were analyzed for statistical significance using ANOVA for a factorial experimental design. The multicomparison significance level for the ANOVA was 0.05. If significance was achieved by one-way analysis, post-ANOVA comparison of means was performed using Tukey's test [36].

## Results and Discussion

### Microarray profiling of TLR expression in airway epithelia

**Figure 1:**
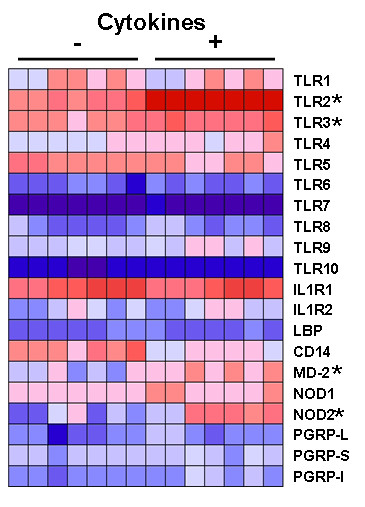
**Heatmap of microarray expression profiling in human airway epithelia following 24 hour stimulation with cytokines (IL-1β (100 ng/ml), TNF-α (100 ng/ml), and IFN-γ (100 ng/ml)) or control**. Represented are expression profiles for microbial pattern recognition proteins including TLRs 1-10, NOD1, NOD2, and the PGRP family members. Also included are the IL-1 receptor variants 1 and 2 and proteins involved in endotoxin sensing (LBP, CD14, and MD-2). Dark blue signifies lowest expression, and dark red denotes highest expression levels. Asterisks indicate genes with significant cytokine induced increases in expression.

We performed a screening microarray expression analysis to profile responses of human airway epithelia to a cocktail of first response pro-inflammatory cytokines. Well-differentiated primary epithelia from seven donors were stimulated with IL-1β (100 ng/ml), TNF-α (100 ng/ml), and IFN-γ (100 ng/ml) for 24 hours. Following treatment, total RNA isolation, preparation of cDNA, and microarray hybridization were performed as described in Materials and Methods. Figure 1 summarizes the expression of selected genes involved in microbial pattern recognition and related responses in airway epithelia under resting vs. cytokine-stimulated conditions. The TLRs were differentially expressed, with TLRs 1 through 5 being most abundant in both resting and cytokine-stimulated airway epithelia. Compared to other TLRs expressed in epithelia, TLR2 expression was markedly (over 8-fold) induced by cytokine stimulation (*P *< 10^-6^) and TLR3 increased ~3-fold. Other genes with notable induction included MD-2 and NOD2, which exhibited over 3-fold and nearly 6-fold increases, respectively. To confirm and further investigate the functional consequences of TLR2 induction, several experiments were performed.

### Induction of TLR2 mRNA expression in airway epithelia

TLR2 expression was previously documented in human airway cell lines, passaged cells in submersion culture, or in lung tissues [2,4,9,10,37]. Although these studies noted TLR2 expression in several model systems, the functional consequences of combined cytokine and glucocorticoid treatment were not investigated, nor were the polarity of TLR2 responses. We focused on functional TLR2 responses in well-differentiated primary airway epithelia. With the exception of studies by Becker et al [37] and Hertz and colleagues [4], TLR2 expression and function has been little studied in this model that closely mimics the *in vivo *airways.

**Figure 2:**
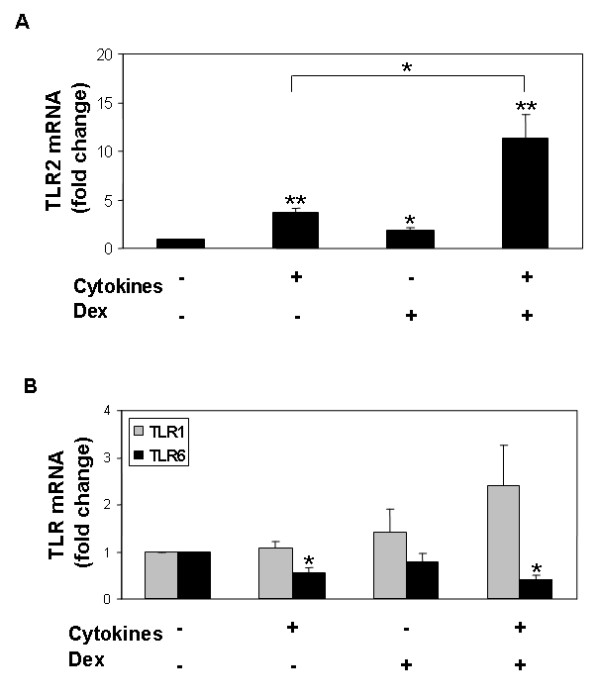
**A) Effects of cytokines and dexamethasone (Dex) on TLR2 mRNA expression**. Cells were treated overnight with cytokines (TNF-α (100 ng/ml) and IFN-γ (100 ng/ml)), dexamethasone (1 μM), or combination of cytokines plus dexamethasone. Quantitative RT-PCR results are represented as fold change from the control (untreated) condition. Data are presented as the mean ± SEM of experiments on 6 donor samples. *P < 0.05, **P < 0.01.** B) Effects of cytokines and dexamethasone (Dex) on TLR1 and TLR6 mRNA expression.** Epithelia were treated as described in Figure 2A. Results of quantitative RT-PCR presented as fold change from control (untreated) condition for TLR1 mRNA (grey) and TLR6 mRNA (black). Data are presented as mean ± SEM from 4 donor samples. *P < 0.05.

We used quantitative RT-PCR to investigate the effects of cytokine and dexamethasone exposure on TLR2 mRNA expression in well-differentiated airway epithelia. Because two recent publications reported the synergistic effects of TNF-α, IFN-γ, and corticosteroids on TLR2 function in airway cell lines [3,24], we focused our subsequent analysis on these stimuli. As shown in Figure 2A, cytokine treatment (TNF-α (100 ng/ml) and IFN-γ (100 ng/ml)) overnight resulted in a 4-fold increase in TLR2 mRNA (*P *< 0.01). A 2-fold increase was seen following treatment with dexamethasone alone (1 μM) (*P *< 0.05), and a 12-fold increase in TLR2 mRNA expression following treatment with cytokines plus dexamethasone (*P *< 0.01). The combination of cytokines plus dexamethasone synergistically enhanced TLR2 mRNA abundance over treatment with cytokines alone (*P *< 0.05).

As cell-surface TLR2 exists as a heterodimer with either TLR1 or TLR6 [21,22], we next investigated the effects of cytokine and dexamethasone exposure on TLR1 and TLR6 mRNA expression. As shown in Figure 2B, TLR1 mRNA abundance remained unchanged following cytokine ± dexamethasone treatment. Unlike TLR2, TLR6 mRNA decreased modestly following cytokine treatment, both in the presence, and absence, of dexamethasone (*P *< 0.05).

### Treatment with cytokines ± dexamethasone alters effects of TLR2 receptor ligation in airway epithelia

To determine the functional effects of increased TLR2 expression, epithelia pre-treated with cytokines ± dexamethasone were stimulated with the TLR1/2 ligand Pam3CSK4 for 24 hours. Two different concentrations of Pam3CSK4, 10 and 25 μg/ml, were studied initially. As greater changes in IL-8 abundance were seen with 25 μg/ml (data not shown), this concentration was used for further studies.

**Figure 3:**
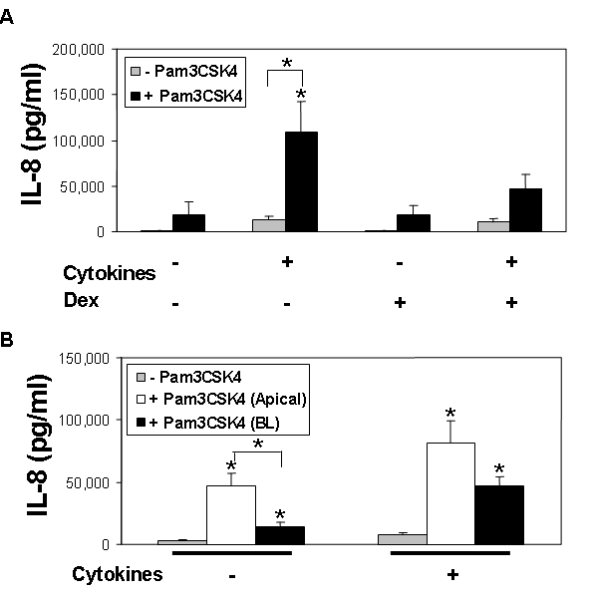
**A) Effects of cytokines and dexamethasone (Dex) on TLR2-mediated IL-8 production**. Epithelia were pre-treated overnight (18 hr) with cytokines (TNF-α and IFN-γ), dexamethasone, or a combination of cytokines plus dexamethasone, then exposed to Pam3CSK4 (25 μg/ml) (black), or control serum-free media (grey) for a period of 24 hours. Data represent the mean ± SEM from 5 donor samples. *P < 0.05. **B) Polarity of TLR2 responses in airway epithelia.** Epithelia were pre-treated overnight (18 hr) with cytokines (TNF-α and IFN-γ), then exposed to Pam3CSK4 (25 μg/ml), applied to either the apical (white) or basolateral (BL, black) surface, or control serum-free media (shown in grey) for a period of 24 hours. Data represent the mean ± SEM from 5 donor samples. *P < 0.05.

Following a 24 hour exposure to the TLR2 ligand, IL-8 increased approximately 9-fold over baseline in cytokine pre-treated epithelia (*P *< 0.05) (Figure 3A). A similar trend toward increased IL-8 was seen in both control cells (*P *= 0.07), and cells pre-treated with cytokines plus dexamethasone (*P *= 0.08). Notably, mean IL-8 production in epithelia pre-treated with cytokines plus dexamethasone was less than half that of cells pre-treated with cytokines alone.

Since epithelia may preferentially express proteins on either their apical or basolateral membrane domains, we asked whether cells exhibited polarized responses to Pam3CSK4. As shown in Figure 3B, IL-8 abundance increased following either apical or basolateral Pam3CSK4 application. In control cells, mean IL-8 production was over 3-fold higher following apical, compared to basolateral application (*P *< 0.05). A similar trend was seen in cytokine pre-treated cells, but did not reach statistical significance. These functional data suggest that TLR2 protein abundance is greater on the apical surface of polarized airway epithelia; however airway epithelia can respond to a TLR2 ligand from either surface.

The changes in TLR2 mRNA abundance were similar in pattern, though less pronounced, than those reported in epithelial cell lines. Prior studies in BEAS-2b [3] and A549 [24] cells demonstrated similar increases in TLR2-mediated IL-8 production following pre-treatment with cytokines plus dexamethasone, but did not compare findings to pre-treatment with cytokines alone. While we detected TLR2 protein in cell lines overexpressing the protein, we were unable to conclusively localize TLR2 protein expression in primary cells using commercially available antibodies and methods including immunohistochemistry and surface biotinylation (data not shown). We conclude that TLR2 protein is functionally present in this model but below the limits of immunodetection.

### Effects of Cytokines on Pam3CSK4 Signaling Responses

Human airway epithelial cells respond to cytokines and bacterial products through induction of specific signaling pathways. The functional expression of TLR2 may be regulated by more than one receptor-mediated signaling pathway prominent in the early responses to bacterial and viral pathogens [9]. Several groups have studied the mechanisms by which dexamethasone enhances TLR2 expression. Glucocorticoids synergistically enhanced nontypeable *Haemophilus influenzae*-induced TLR2 expression via induction of MAPK phosphatase-1, leading to inhibition of p38 MAPK [38]. The results of Hermoso et al [24] suggest that TNF-α and dexamethasone cooperatively regulate the TLR2 promoter, through the involvement of NF-kB and STAT transcription factors, as well as a 3'-glucocorticoid response element.

**Figure 4:**
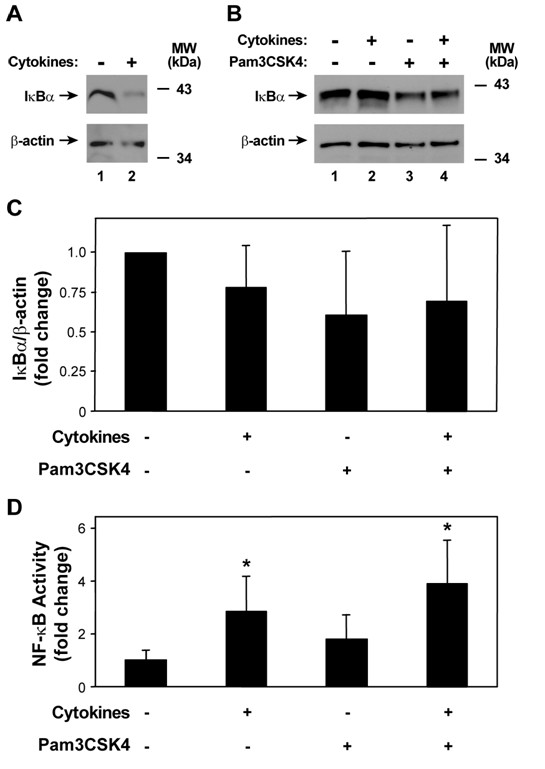
**Cytokine-induced NF-κB activation is minimally augmented during subsequent TLR2 stimulation**. A) IκBα and β-actin cellular protein levels were assessed using immunoblot analysis of extracts from human airway epithelia that were treated without or with media containing TNF-α (100 ng/ml) and IFN-γ (100 ng/ml) for 30 min. B) IκBα and β-actin cellular protein levels were assessed using immunoblot analysis of extracts from human airway epithelia that were first treated without or with media containing TNF-α (100 ng/ml) and IFN-γ (100 ng/ml) for 24 hours, followed by incubation without or with Pam3CSK4 (25 mg/ml) for 30 min. C) IκBα and β-actin protein levels in the experiment outlined in *B *were quantified using band densitometry of immunoblot analyses results with inclusion of samples from three individuals. Values were calculated as IκBα/β-actin, were normalized to the untreated control value for each individual, and are expressed as mean fold change in IκBα ± S.D. (*n *= 3 in each group). D) NF-κB-dependent gene activation was assessed using luciferase activity assays of extracts from human airway epithelia that were initially infected with an adenoviral vector expressing a luciferase gene driven by four tandem NF-κB sites. Cells were then treated without or with media containing TNF-α (100 ng/ml) and IFN-γ (100 ng/ml) for 24 hours, followed by incubation with Pam3CSK4 (25 mg/ml) for 24 hours. Values are expressed as mean ± S.D. (*n *= 2-3 samples from 4 individuals in each group), and a significant difference from the untreated control is indicated by an *asterisk*.

In these experiments we investigated signaling in response to conditions involving cytokine pre-treatment and TLR2 ligand application, as this setting was associated with the maximal responses to TLR2 ligand treatment. We initially assumed that cytokine augmentation of TLR2-mediated responses would coincide with increased nuclear factor-κB (NF-κB) pathway activation because this transcription factor is critical for regulation of the expression of many host defense genes [35,39]. Under basal conditions, epithelial and other cells sequester NF-κB family members in the cytoplasm bound to inhibitor of κB (IκB) proteins [40]. A variety of stimuli, including bacterial molecules and host mediators, induce serine phosphorylation of IκB proteins, thereby targeting them for ubiquitination. These modifications of IκB lead to 26S proteasome degradation, resulting in NF-κB release and translocation to the cell nucleus where it mediates defense gene activation [41]. To assess NF-κB pathway activation, we initially used immunoblot analyses to identify IκB degradation. As expected, the combination of TNF-α and IFN-γ for 30 minutes decreased IκBα in epithelia (Figure 4A), but this effect did not appear to persist after 24 hours of exposure followed by 30 minutes without treatment (Figure 4B). In addition, pretreatment with the cytokine mixture did not clearly affect IκBα levels in epithelia after TLR2 receptor stimulation (Figure 4C). Assessment of NF-κB-dependent gene activation using an adenoviral vector expressing a luciferase gene driven by four tandem NF-κB sites revealed no cytokine augmentation of TLR2-mediated NF-κB activation that correlated with the observed increased inflammatory gene expression, although there was significant variability between samples (Figure 4D). Increased NF-κB activity was observed after cytokine treatment of epithelia. Thus, augmentation of NF-κB does not seem to account for cytokine pretreatment effects on TLR2-induced gene expression.

**Figure 5:**
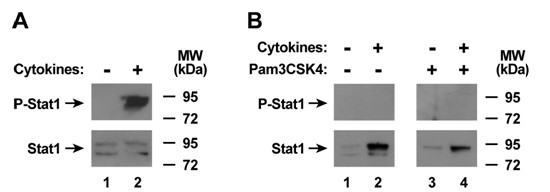
**Cytokine-induced Stat1 activation does not persist during subsequent TLR2 stimulation**. A) Phosphorylated and total Stat1 cellular protein levels were assessed using immunoblot analysis of extracts from human airway epithelia that were treated without or with media containing TNF-α (100 ng/ml) and IFN-γ (100 ng/ml) for 30 min. B). Phosphorylated and total Stat1 cellular protein levels were assessed using immunoblot analysis of extracts from human airway epithelia that were first treated without or with media containing TNF-α (100 ng/ml) and IFN-γ (100 ng/ml) for 24 hours, followed by incubation without or with Pam3CSK4 (25 mg/ml) for 30 min.

**Figure 6:**
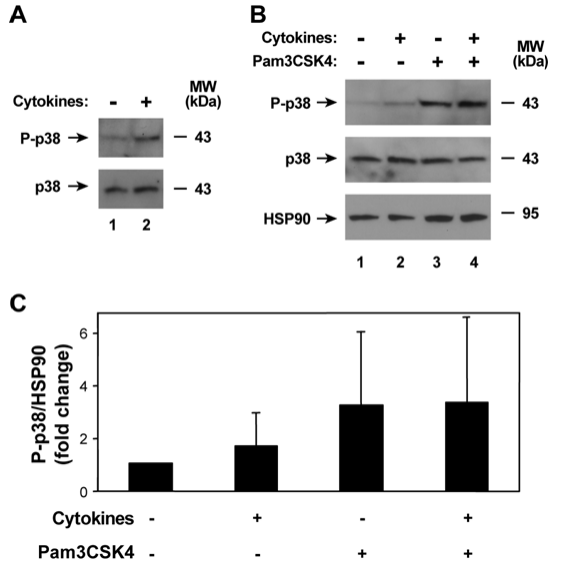
**Cytokine-induced p38 MAP kinase activation does not augment subsequent TLR2 stimulation**. A) Phosphorylated and total p38 cellular protein levels were assessed using immunoblot analysis of extracts from human airway epithelia that were treated without or with media containing TNF-α (100 ng/ml) and IFN-γ (100 ng/ml) for 30 min. B) Phosphorylated and total p38, and heat shock protein-90 (HSP90) cellular protein levels were assessed using immunoblot analysis of extracts from human airway epithelia that were first treated without or with media containing TNF-α (100 ng/ml) and IFN-γ (100 ng/ml) for 24 hours, followed by incubation without or with Pam3CSK4 (25 mg/ml) for 30 min. C) Phosphorylated p38 and HSP90 protein levels in the experiment outlined in *B *were quantified using band densitometry of immunoblot analyses results with inclusion of samples from three individuals. Values were calculated as phosphorylated p38/HSP90, were normalized to the untreated control value for each individual, and are expressed as mean fold change in phosphorylated p38 ± S.D. (*n *= 3 in each group).

We also examined other pathways that affect host defense gene expression in human airway epithelia. IFN-γ-induced effects in epithelial and other cells requires activation of the transcription factor Stat1 by phosphorylation of tyrosine-701, with subsequent nuclear translocation and binding to gamma interferon activation sites in IFN-γ-responsive genes [42,43]. The combination of TNF-α and IFN-γ for 30 minutes induced Stat1 phosphorylation (Figure 5A), but this effect did not persist after 24 hours of exposure followed by 30 minutes without or with treatment with the TLR2 agonist (Figure 5B). Another pathway that modifies inflammatory gene expression in epithelial and other cells is the p38 mitogen-activated protein (MAP) kinase pathway [33]. Activation of p38 also occurs through phosphorylation, and the combination of TNF-α and IFN-γ for 30 minutes increased the level of phosphorylated p38 in epithelia (Figure 6A). This effect was more pronounced in epithelia treated with the TLR2 agonist, but no difference was noted without or with pretreatment with the cytokine mixture (Figures 6B and 6C). Based on these results, augmentation of TLR2-induced host defense gene expression with TNF-α and IFN-γ pretreatment does not appear to be due to modulation of these three pathways (at least at the time points investigated), but likely is through other mechanisms, or combinatorial effects not assessed by these assays.

### TLR2 receptor ligation enhances expression of inducible host defense proteins and increases antimicrobial activity in airway surface liquid

**Figure 7:**
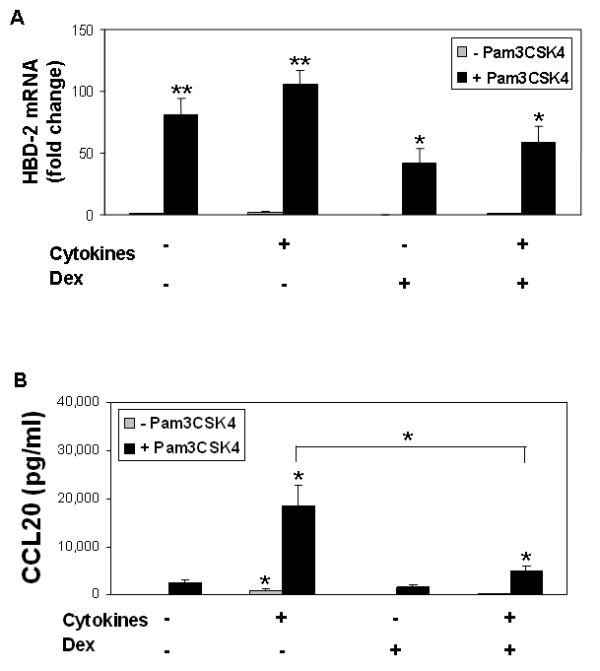
**A) Effects of cytokines and dexamethasone (Dex) on TLR2-mediated HBD-2 expression**. Epithelia were pre-treated overnight (18 hr) with cytokines (TNF-α and IFN-γ), dexamethasone, or a combination of cytokines plus dexamethasone, then exposed to Pam3CSK4 (25 μg/ml) (black), or control serum-free media (grey), for a 24-hour period. HBD-2 mRNA abundance determined by quantitative PCR. Data represent mean ± SEM from 5 donor samples. *P < 0.05, **P < 0.01. **B) Effects of cytokines and dexamethasone (Dex) on TLR2-mediated CCL20 release.** Primary cultures were pre-treated overnight with cytokines (TNF-α and IFN-γ), dexamethasone, or combination of cytokines plus dexamethasone, then exposed to Pam3CSK4 (25 μg/ml) (black), or control serum-free media (grey) for a period of 24 hours. Data represent mean ± SEM from 5 donor samples. *P < 0.05.

We further hypothesized that TLR2 engagement would activate expression of inducible host defense proteins. We examined expression of the antimicrobial peptide HBD-2 [23], a known product of airway epithelia in response to TLR2 agonist exposure [4]. Primary cultures of human airway epithelia were pre-treated with cytokines ± dexamethasone, then stimulated with Pam3CSK4 for 24 hours. As shown in Figure 7A, HBD-2 mRNA abundance remained low in unstimulated cultures, even following cytokine pre-treatment. After exposure to the TLR2 agonist HBD-2 mRNA expression increased significantly in all conditions. However, when contrasted with unstimulated or cytokine treated cells, the increase in HBD-2 mRNA was significantly less in epithelia pre-treated with dexamethasone or cytokines plus dexamethasone.

We also assayed expression of CCL20, a peptide with both innate and adaptive immune functions produced by airway epithelia. CCL20 stimulates B-cell migration and has antimicrobial activity comparable to the β-defensins [44]. Following Pam3CSK4 stimulation, CCL20 increased 25- to 30-fold over baseline in cells pre-treated with cytokines (Figure 7B). Dexamethasone significantly blunted this response, as CCL20 abundance in epithelia pre-treated with cytokines plus dexamethasone was approximately one-third that of cells treated with cytokines alone (*P *< 0.05).

**Figure 8:**
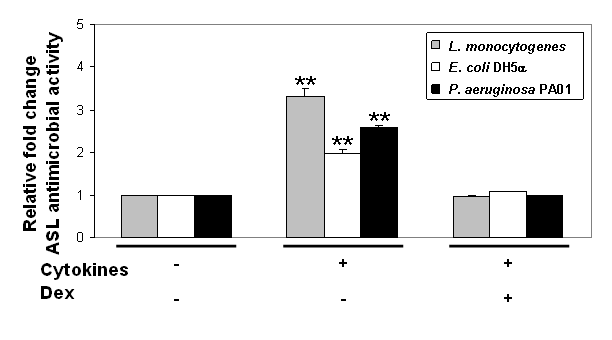
**TLR2-mediated ASL antimicrobial activity**. Epithelia were pre-treated overnight with cytokines (TNF-α and IFN-γ), dexamethasone, or a combination of cytokines plus dexamethasone, then exposed to Pam3CSK4 (25 μg/ml) for 24 hours. Apical washings were obtained, and modified radial diffusion assays performed. Data represent fold change from control, TLR2-agonist exposed condition for the test organisms: *Listeria monocytogenes *(grey), *Escherichia coli *DH5α (white), and *Pseudomonas aeruginosa *PA01 (black). *n *= 3 donor samples. **P < 0.01.

We next investigated the effects of cytokine and dexamethasone exposure on TLR2-mediated airway surface liquid (ASL) antimicrobial activity. As shown in Figure 8, ASL antimicrobial activity from epithelia exposed to the TLR2 agonist increased significantly following cytokine pre-treatment, compared to control, for each of the 3 organisms studied (*Listeria monocytogenes, Escherichia coli *DH5α, and *Pseudomonas aeruginosa *PA01). In contrast, net antimicrobial activity from cells pre-treated with cytokines plus dexamethasone was unchanged from control. Antimicrobial activity was also unchanged following pre-treatment with dexamethasone alone (data not shown). In the absence of TLR2 agonist exposure, ASL antimicrobial activity was unaltered by exposure to cytokines ± dexamethasone. These results provide further evidence of decreased TLR2-mediated innate immune responses in the setting of combined exposure to cytokines plus dexamethasone. Furthermore, these data suggest that both pro-inflammatory cytokines and a TLR2 stimulus synergistically enhanced TLR2 function in airway epithelia. Together, these findings suggest that TLR2 function in cytokine-exposed human airway epithelia is diminished, rather than augmented, by dexamethasone, despite increases in TLR2 mRNA abundance.

## Conclusion

We conclude that TLR2 is an inducible component of airway epithelial defenses. Enhanced functional TLR2 expression following TNF-α and IFN-γ exposure may serve as a dynamic means to amplify innate immune responses during infectious or inflammatory pulmonary diseases. Under conditions where first response cytokines are present, enhanced TLR2 signaling allows for further amplification of mucosal immunity. This increase in signalling does not correlate well with NF-κB, Stat1, or p38 MAP kinase pathway activation. The importance of lipopeptide recognition in airway defense is further demonstrated by the upregulation of several host defense proteins/peptides following receptor engagement. Our finding that glucocorticoids can act as a negative regulator of functional TLR2 expression in well-differentiated human airway epithelia has clinical implications in settings of systemic or inhaled corticosteroid use. Since TLR2-mediated responses may occur early in the host response to infection, any factors that negatively impact TLR2 expression or signaling might influence disease outcomes.

## Competing interests

The authors declare that they have no competing interests.

## Authors' contributions

AAW, BNN, LJM, CW-L carried out the experimental work, the data analysis, and drafted the manuscript. TES and DCL participated in the experimental work. AAW participated in the design of the study. PBM and DCL conceived the hypotheses, advised on experimental work and assisted in drafting the manuscript.
